# Observable and Unobservable Mechanical Motion

**DOI:** 10.3390/e22070737

**Published:** 2020-07-03

**Authors:** J. Gerhard Müller

**Affiliations:** Department of Applied Sciences and Mechatronics, Munich University of Applied Sciences, D-80335 Munich, Germany; gerhard.mueller@hm.edu

**Keywords:** mechanical motion, energy dissipation, information gain, Hamilton’s equations of motion, principle of least action

## Abstract

A thermodynamic approach to mechanical motion is presented, and it is shown that dissipation of energy is the key process through which mechanical motion becomes observable. By studying charged particles moving in conservative central force fields, it is shown that the process of radiation emission can be treated as a frictional process that withdraws mechanical energy from the moving particles and that dissipates the radiation energy in the environment. When the dissipation occurs inside natural (eye) or technical photon detectors, detection events are produced which form observational images of the underlying mechanical motion. As the individual events, in which radiation is emitted and detected, represent pieces of physical action that add onto the physical action associated with the mechanical motion itself, observation appears as a physical overhead that is burdened onto the mechanical motion. We show that such overheads are minimized by particles following Hamilton’s equations of motion. In this way, trajectories with minimum curvature are selected and dissipative processes connected with their observation are minimized. The minimum action principles which lie at the heart of Hamilton’s equations of motion thereby appear as principles of minimum energy dissipation and/or minimum information gain. Whereas these principles dominate the motion of single macroscopic particles, these principles become challenged in microscopic and intensely interacting multi-particle systems such as molecules moving inside macroscopic volumes of gas.

## 1. Introduction

The basis of analytical mechanics was laid in the 18th century by Joseph Louis Lagrange and by Sir William Rowan Hamilton. Lagrange’s and Hamilton’s equations of motion [1,2] successfully describe the mechanical motion of macroscopic particles with the planetary motion in the solar system being a celebrated showcase. As Lagrange’s and Hamilton’s equations of motion are invariant under the time reversal operation t→−t, their solutions describe time-reversible modes of mechanical motion. Later, in the 19th century, when the foundations of thermodynamics and of the kinetic theory of gases were developed, this time-reversibility appeared as a mental stumbling block to researchers such as Clausius, Boltzmann, Lord Kelvin, and Maxwell. Trying to reconcile the irreversible behavior of gases with the intrinsic reversibility of the mechanical motion of its molecular constituents, Maxwell formulated his sorting demon paradox which soon became famous under the name “Maxwell’s demon paradox” [3]. Sticking to the belief that mechanical motion is time-reversible also on a molecular scale, it was claimed that a sorting demon acting inside a gas should be able to create temperature or pressure differences without expending own work and, therefore, be able to extract useful mechanical work from a single reservoir, in obvious contradiction with the second law of thermodynamics [4,5]. Ever since its invention in 1871, this paradox attracted the interest of researchers and caused them to propose different kinds of explanations up to the present time. A good account of these historical developments can be found in the books of Leff and Rex [6,7].

The subject matter of the present paper relates to this problem of reversibility of mechanical motion. The point we should like to make here is that analytical mechanics implicitly deals with the mental abstraction of unobserved and unobservable mechanical motion, whereas any kind of observable motion entails more or less pronounced degrees of irreversibility which are connected with its observational process.

**Figure 1:**
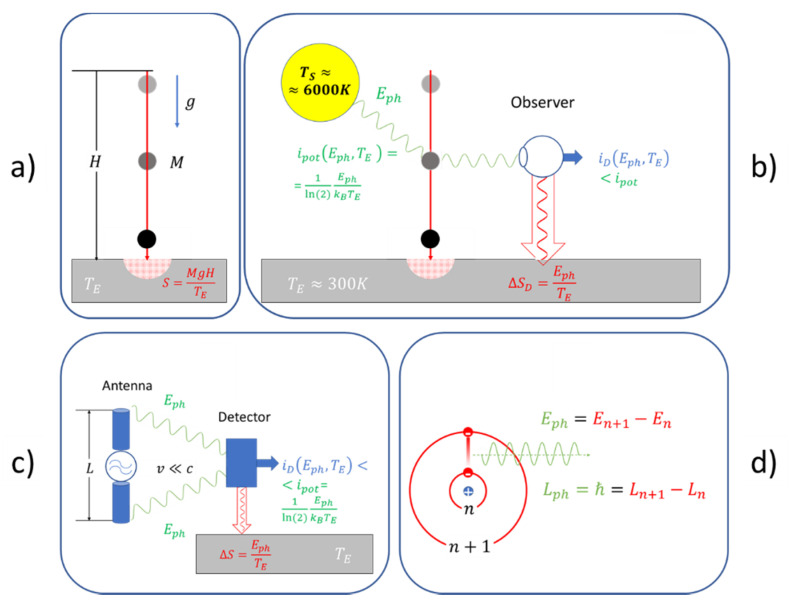
(**a**) Particle dropping to ground without any observations being made; (**b**) particle dropping to ground with sunlight being scattered into the eye of an observer; (**c**) electron moving up and down a center-fed antenna producing pulses of radiation which can be detected at a distance; (**d**) emission of a photon from an atom with the photon carrying away signatures ($E_{ph}$, ħ) of the electronic transition to some remote point of detection.

In order to illustrate our ideas, consider Figure 1. While the top panels (Figure 1a,b) relate to trivial cases of everyday mechanical motion, the bottom panels (Figure 1c,d) relate to the mechanical motion of electrons, i.e., electrically charged particles.

Turning to the top panel first, Figure 1a sketches a particle of mass M dropping from a height H to the ground at H=0. As long as the particle is freely falling through empty space, its mechanical motion is adequately described by the time-reversible Hamilton’s equations. As no interaction with a measurement instrument does take place during this travel, the time evolution of the trajectory cannot be followed and meaningful comparisons with the predictions of theory cannot be made. The only effect that is observable is the impact of the particle on the ground when its kinetic energy is converted into low-temperature heat at the environmental temperature $T_{E}$. Measuring the dissipated energy, the speed of the particle at H=0 can be determined and a test of the time-reversibility of Hamilton’s equations, in principle, could be made by re-converting the dissipated energy into kinetic energy and by sending the particle back to its initial height H and checking whether the same time is required for the travel back up. As, due to the second law of thermodynamics, a re-conversion of dissipated energy into kinetic energy is impossible, Figure 1a clearly represents an unobserved and unobservable kind of mechanical motion.

Figure 1b, in contrast, shows an observed version of this same example. Here, the sun illuminates the falling particle which causes some of the sun’s photons with energy $E_{ph}\gg k_{B}T_{E}$ to become scattered into the eye of an observer, who can track the trajectory of the falling particle by performing repeated “measurements”. All these measurements very obviously involve irreversibility as inside the eye the energy of the scattered photons is dissipated and converted into low-temperature heat. Whereas such dissipation also happens to all other photons which become scattered at the falling particle and which directly hit the earth’s surface, energy dissipation inside the eye transiently converts some of the absorbed photon energy into an electrochemical output signal that can be communicated to the brain for further informational processing. We showed in a recent publication that, during such a detection, a photon detector, like the human eye, effectively performs as a thermodynamic engine that partly converts potential information, $i_{pot}\left(E_{ph},T_{E}\right)=\frac{1}{\ln\left(2\right)}\frac{E_{ph}}{k_{B}T_{E}}$, carried with the sun’s photons themselves, into information $i_{D}\left(E_{ph},T_{E}\right)<i_{pot}\left(E_{ph},T_{E}\right)$ that is realized in the detection process [8,9]. Although this second example demonstrates the irreversibility of the observation process, it also suggests that observation might be considered a secondary process that occurs independently of the mechanical motion and that only takes place when the sun is shining and when an observer happens to be in place. Further considering the energetically and entropically tiny magnitude of the observation process, these latter considerations show that the 18th century founders of analytical mechanics could rightfully ignore the role of observation in their description of mechanical motion.

A fact that was not known to the founders of analytical mechanics is that each light–matter interaction occurs with atoms and molecules which are composed of equal amounts of positive and negative electrical charge. Another fact that was unknown to those founders was that electrically charged particles emit bursts of electromagnetic radiation whenever they suffer accelerations or decelerations [10,11]. As, in the case of charged particles, the emission of radiation is directly coupled to their mechanical motion, the observation of charged particles does not require any external sources of energy. As the energy required for the informational process is directly extracted from the motional energy of the moving particles and as the radiation is running away from its sites of emission, a certain level of irreversibility is introduced into the mechanical motion. Figure 1c presents an everyday example of such charged particle motion. There, a linear, center-fed antenna is shown in which electrons are driven up and down across a macroscopic distance L with speeds v much smaller than the speed of light (v≪c). As these vibrating electrons reach their upper and lower turning points, pulses of radiation are emitted which extract mechanical energy from the vibrating electrons and which carry this energy from the sites of emission to a detector which is placed at some distance. Once arrived there, this energy is dissipated and converted into low-temperature heat while intermittently producing macroscopically observable events which represent observational images of the electron motion inside the antenna rod. Whereas, in such macroscopic arrangements, only very tiny amounts of the motional energy are carried away in the form of electromagnetic radiation, the effects of radiation damping become increasingly more important as the amplitude L of vibration is reduced and as the speed v of vibration is increased. In particular, when spatio-temporal domains of the size of atoms and molecules are reached, the emission occurs in the form of photons which carry discrete amounts of energy $E_{ph}$ and angular momentum ħ from their sites of emission to potential sites of detection (Figure 1d). Moreover, as the photon energies and photon angular momenta are signatures of those changes in mechanical motion that caused their emission, photons obviously carry potential information that may be turned into realized information once these become detected at some remote location. Photon emission and photon detection, therefore, are key processes through which mechanical motion becomes detectable, albeit at the expense of energy dissipation.

Building on the above considerations, we present herein a thermodynamic approach to mechanical motion which explicitly takes into account those dissipative effects that are connected with the radiation damping and the conversion of the emitted radiation into observations. In the course of our discussion, we develop the idea that the mechanical motion of charged particles is burdened with an observational overhead that can be measured in terms of physical action generated and energy dissipated during the processes of radiation emission and radiation detection. Studying examples of macroscopic mechanical motion, we show that the principle of least action, which lies at the heart of Hamilton’s equations of motion [1,2], not only minimizes the physical action that is associated with the particle motion itself but that it also minimizes those physical overheads that are connected with the radiation damping and the observation of the particle motion. Arguing from the standpoint of thermodynamics and information theory, the principle of least action, therefore, appears as a principle of minimum energy dissipation and/or minimum information gain. Whereas, in the specific case of rectilinear mechanical motion under zero force conditions, the observational overhead vanishes, and the mechanical motion becomes ideally reversible, but also unobservable, the principles of least action, and least dissipative and observational overheads become challenged when the particles under consideration are swiftly moving and intensely interacting particles, as for instance, molecules moving inside a macroscopic volume of gas. In this latter case, very small but finite, dissipative overheads are burdened onto the mechanical motion.

## 2. Thermodynamic Approach to Classical Mechanical Motion

The thermodynamic approach of describing physical systems considers the way in which such systems are able to exchange energy with their environments. As energy can manifest itself in many different forms, such exchanges can proceed in a variety of ways. Quantitatively, this fact is expressed by differential forms of the kind

(1) $$dE=\sum_{i}\xi_{i}d\eta_{i},$$

which are commonly known as Gibbs fundamental forms [4,12,13]. Here, dE is the total amount of energy exchanged, while the products $\xi_{i}d\eta_{i}$ of intensive ($\xi_{i}$) and extensive variables ($\eta_{i}$) stand for the individual forms in which such energy exchanges can take place.

The kinds of systems that are customarily considered in thermodynamics usually take on forms such as

(2) $$dE=TdS-pdV+\overset{n}{\underset{i=1}{\sum }}\mu_{i}dN_{i}.$$

Such fundamental forms describe changes in the system’s internal energy through exchanges of heat (TdS), mechanical work (pdV), and particles ($\mu_{i}dN_{i}$) with the environment. A subset of such problems relates to chemistry, where a set of different particles is confined to the interior of a volume V, which might or might not expand while being maintained at a temperature T. The sums $\sum_{i=1}^{n}\mu_{i}dN_{i}$ then describe chemical reactions between the different kinds of particles enclosed in the volume V and represented by particle numbers $N_{i}$ and chemical potentials $\mu_{i}$.

As described in detail in the textbooks of Falk and Ruppel [14,15], such Gibbs fundamental forms are also able to deal with situations not conventionally treated in thermodynamics. A particular example is the motion of point-like particles of mass M moving in conservative force fields. The fundamental forms dealing with such cases are

(3) dE=vdP−Fdr,

where the bold characters stand for the vectors of the dynamical velocity $v=\frac{dE}{dP}=\left[\frac{dE}{dP_{x}},\frac{dE}{dP_{y}},\frac{dE}{dP_{z}}\right]$, with P standing for the linear momentum of the particle and F(***r***) for the force acting on this particle at the location r as it moves along its trajectory inside the system.

In case the particle does not exchange energy with external systems, the system’s internal energy E(P,r,t) remains constant,

(4) dE=vdP−Fdr=0

and its time rate of change vanishes,

(5) $$\frac{d}{dt}E\left(P,r,t\right)=v\left(r,t\right)\frac{d}{dt}P\left(r,t\right)-F\left(r\right)\frac{d}{dt}r\left(t\right)=0.$$

From this latter equation, it follows that

(6) $$\frac{d}{dt}r\left(t\right)=v\left(r,t\right)$$

and

(7) $$\frac{d}{dt}P\left(r,t\right)=F\left(r,t\right).$$

Whereas the first of these conditions implies that the kinetic velocity $\frac{d}{dt}r\left(t\right)$ needs to be equal to the dynamical velocity $v\left(r,t\right)=\frac{dE\left(r,\;t\right)}{dP}$ of the particle, the second states that the time rate of change of the particle momentum needs to match the force acting on the particle at the point r of its trajectory. Both conditions, obviously, reduce to Hamilton’s equations of mechanical motion if one puts E(P,r, t)=H(P,r, t) [1,2,14].

**Figure 2:**
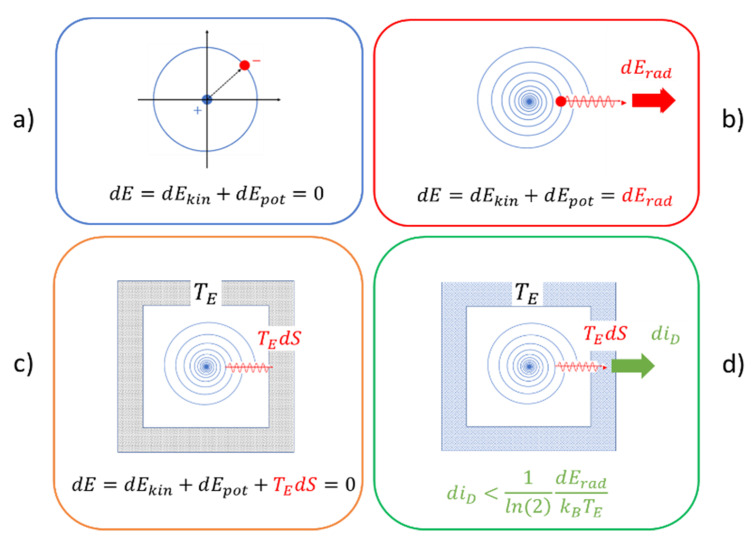
(**a**) Circular motion of a charged particle in a conservative central force field; (**b**) same process as in (**a**) but allowing for radiation emission from accelerated charges; (**c**) same process as in (**b**) with the emitted radiation being absorbed in a macroscopic heat reservoir of temperature $T_{E}$, thereby producing an amount of entropy $dS=\frac{dE_{rad}}{T_{E}}$ and an increase $d\left(mi\right)=\frac{1}{\ln\left(2\right)}\frac{dE_{rad}}{k_{B}T_{E}}$ in missing information concerning the internal state of motion inside the reservoir walls; (**d**) absorption of radiation in a radiation detector producing a piece of macroscopically observable information that is accessible to outside observers and providing an information gain $di_{D}<d\left(mi\right)$.

Such a process of conservative motion is pictorially presented in Figure 2a, considering the specific case of circular motion in a conservative central force field. Assuming that the moving particle does not carry any electrical charge, no radiation is emitted, and the kinetic and potential energies of the particle remain constant over time, i.e.,

(8) $$dE=vdP-Fdr=\omega dL-Dd\varphi =dE_{kin}+dE_{pot}=0.$$

In this latter equation, φ is the turning angle, ω is the angular velocity, L is the angular momentum, and D is the torque acting on the rotating particle.

When the particle does carry an electrical charge, as assumed in Figure 2b, electromagnetic radiation is emitted, and energy is carried away from the moving particle. For reasons of energy conservation, the particle then has to move closer to its center of rotation, thus reducing its potential energy and increasing its kinetic energy. The energy balance equation then takes on the form

(9) $$dE=dE_{kin}+dE_{pot}=dE_{rad},$$

with $dE_{rad}$ standing for the energy emitted in the form of electromagnetic radiation. As this process continues with time, the orbiting particle spirals inward toward its center of rotation, as shown in Figure 2b. As the emitted radiation never returns, it becomes evident that radiative energy loss is the principle and unavoidable dissipative mechanism that accompanies the classical motion of charged particles.

Once emitted, the radiation normally becomes absorbed by macroscopic pieces of matter, in which the radiation energy is internally dissipated and turned into low-temperature heat. Arguing within the Gibbs approach, the open system of Figure 2b can be turned back into a closed system by representing the absorbing matter with solid walls that surround the orbiting particle and that behave like macroscopic heat reservoirs maintained at the environmental temperature $T_{E}$. In this latter case, shown in Figure 2c, the Gibbs fundamental form turns into

(10) $$dE=dE_{kin}+dE_{pot}+T_{E}dS=0,$$

with $T_{E}dS=dE_{rad}$ standing for the thermal energy generated inside the reservoir walls. In contrast to Equation (8), which describes a case of conservative motion, Equation (10) describes a case of dissipative motion. Once it is assumed that the internal energy U of the surrounding reservoir walls is large compared to the emitted radiation energy, i.e., $U\gg dE_{rad}$, the radiation is absorbed without causing any measurable temperature change. All informational value carried with the emitted radiation is thereby immediately degraded and turned into a deficit, i.e., into an increased amount of missing information, d(mi), concerning the internal state of motion inside the reservoir walls [13]. Formally, this increase in missing information can be expressed as

(11) $$d\left(mi\right)=\frac{1}{\ln\left(2\right)}\frac{dE_{rad}}{k_{B}T_{E}}.$$

So far, all cases considered in Figure 2a–c represent cases of unobservable mechanical motion because no radiation is emitted at all in scenario (a), the radiation disappears in empty space in scenario (b), or because its informational value is directly converted into low-temperature heat in scenario (c). The only way to arrive at a situation of observable mechanical motion is modifying scenario (c) into the one shown in Figure 2d. There, the reservoir walls are replaced by detector walls which again take the form of heat reservoirs as in Figure 2c, but constructed in a way that allows macroscopically observable output signals F(t) to be generated and communicated to outside observers whenever these walls are struck by radiation emitted from within the detector walls [9,16]. The scenario of Figure 2d, therefore, again represents an open system, but one that internally involves energy dissipation and that, in response to the energy dissipation, is able to provide an observational image to outside observers of those mechanical motion processes that are going on within the detector walls.

## 3. Mechanical Motion in the Quantum Domain

When the circular motion of charged particles takes place in spatial dimensions comparable to atomic or molecular sizes, the electromagnetic radiation is emitted in the form of discrete energy packages, i.e., in the form of photons. For the sake of illustration, we consider below the motion of electrons within H-atoms. With the emission of electromagnetic radiation now taking place in the form of discrete photons, the energy balance Equation (9) becomes

(12) $$\Delta E_{mn}=\Delta E_{kin\_mn}+\Delta E_{pot\_mn},$$

with the radiation energy quanta $\Delta E_{mn}$ complying with the Rydberg formula [17,18,19],

(13) $$\Delta E_{mn}=E_{Ryd}\left(\frac{1}{n^{2}}-\frac{1}{m^{2}}\right),$$

(14) $$E_{Ryd}=k_{e}{}^{2}\frac{m_{e}q^{4}}{2\hbar^{2}},$$

in which m and n stand for the orbital quantum numbers of initial and final states, $m_{e}$ and q stand for the mass and charge of the orbiting electron, and ħ stands for Planck´s constant; $k_{e}=1/4\pi \varepsilon_{0}$, finally, is the Coulomb constant.

**Figure 3:**
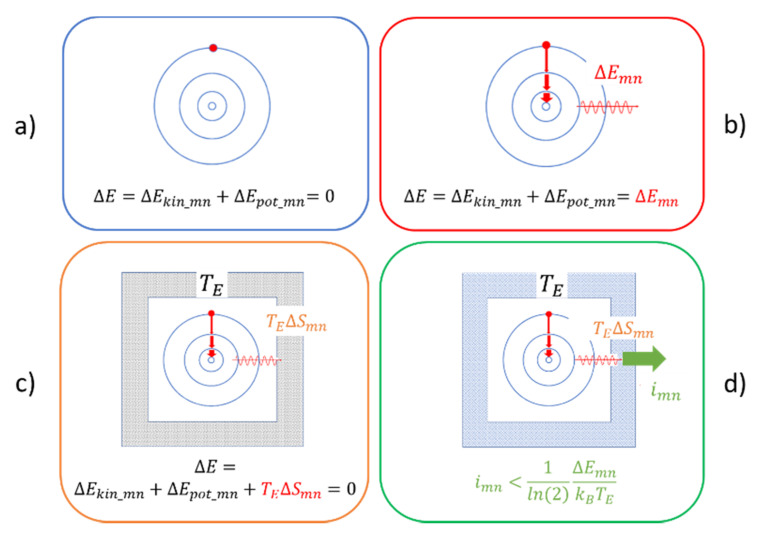
(**a**) Radiation-less motion in an excited Bohr atom; (**b**) same process as in (**a**) but with finite radiative lifetime and emission energies following the Rydberg formula; (**c**) same process as in (**b**) with the emitted radiation being absorbed in a macroscopic heat reservoir of temperature $T_{E}$ ($T_{E}\Delta S_{mn}$ = $\Delta E_{mn}$); (**d**) absorption of emitted radiation in a radiation detector operated at a temperature $T_{E}$.

With these changes having been implemented, the quantum analogues of the classical situations in Figure 2 now look like those in Figure 3. There, Figure 3a–c again represent cases of unobservable mechanical motion, while Figure 3d is the only one that deals with observable motion.

Turning to Figure 3a first, we note that this represents the fictitious case of a closed system in which all changes are internal to the atom. In this situation, any changes in potential energy are balanced by compensating changes in kinetic energy,

(15) $$\Delta E_{kin\_mn}=-\Delta E_{pot\_mn}.$$

As for any choice of quantum numbers m and n energy is conserved, no radiation needs to be emitted. This first case, therefore, represents a case of unobservable mechanical motion, similar to the classical Hamiltonian case in Figure 2a. Moreover, with energy being internally conserved, there is also no driving force that would favor downward transitions to states with lower potential energy over upward transitions to states with higher potential energy. This first case of unobservable motion, therefore, also represents a case of reversible mechanical motion.

**Figure 4:**
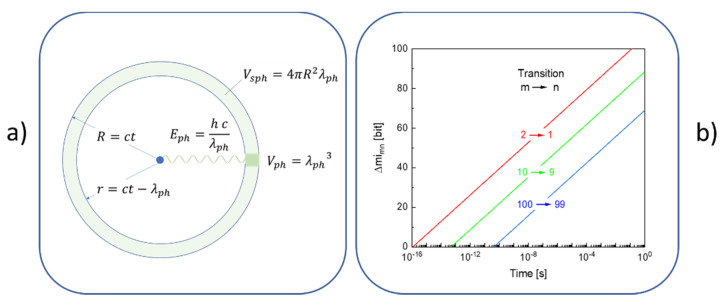
(**a**) Uncertainty in localization of a photon travelling out from the emitting atom in the center; (**b**) resulting loss in locational information as a function of time after photon emission.

Figure 3b illustrates a very different situation. In this latter case, the emission energies of the photons are assumed to match with those given by the Rydberg formula (Equation (14)). As in this case, any loss of potential energy in a downward transition is no longer balanced by an equal gain in kinetic energy, the misbalance in energy needs to be carried away by a photon. Whereas, classically, the emitted radiation would take the form of a spherical wave traveling outward with the speed of light, the emitted photon with energy $\Delta E_{mn}$ and wavelength $\lambda_{mn}=hc/\Delta E_{mn}$ could be anywhere within a spherical shell of radius R=ct and thickness $\lambda_{mn}$ after a time t has elapsed after its emission (see Figure 4a). The resulting uncertainty in photon location can then be expressed as a change in entropy,

(16) $$\Delta S_{mn}\left(t\right)=k_{B}ln\left[V_{sph}\left(t\right)/V_{mn}\right],$$

with $V_{sph}\left(t\right)$ standing for the time-dependent volume of the spherical shell and $V_{mn}=\lambda_{mn}{}^{3}$ for the effective volume of the photon. Alternatively, by converting Equation (16) to information units the corresponding loss of information, $\Delta mi_{mn}\left(t\right)$, concerning the photon localization, becomes

(17) $$\Delta mi_{mn}\left(t\right)=\frac{1}{\ln\left(2\right)}\left[4\pi {\left(\frac{\Delta E_{mn}\;t}{h}\right)}^{2}\right].$$

With the change $\Delta S_{mn}\left(t\right)$ being positive, the change in free energy,

(18) $$\Delta F_{mn}\left(t\right)=\Delta E_{mn}-T_{E}\Delta S_{mn}\left(t\right),$$

of the emitting atom rapidly decreases with time and even becomes negative very shortly after the emission has taken place. The emission of radiation, therefore, is a strong thermodynamic driving force that drives electrons to perform downward transitions to states with lower potential energy. Conversely, upward transitions to states with higher potential energy are strongly discouraged as this would involve transitions with the radiation moving backward in time and toward final states with lower entropy. Figure 3b, therefore, pictures an open system, undergoing irreversible mechanical motion and emitting photons, which carry potential information with regard to the m→n transition, which, however, gets lost as the emitted photons move out toward infinity. Figure 3b, therefore, once again represents a case of unobservable mechanical motion, but one exhibiting dissipation.

Figure 3c illustrates a situation in which the emitted photons are no longer able to travel out to infinity as these become absorbed by pieces of macroscopic matter on their way out. For simplicity, the absorbing matter is represented in Figure 3c by solid walls that surround the radiation-emitting atom and that effectively perform as macroscopic heat reservoirs, maintained at the environmental temperature $T_{E}$. Once absorbed inside this reservoir, the photon energy $\Delta E_{mn}$ is dissipated without affecting any measurable temperature change, and the potential information,

(19) $$i_{mn}=\frac{1}{\ln\left(2\right)}\frac{\Delta E_{mn}}{k_{B}T_{E}},$$

carried with the photons directly after emission is converted into missing information about the internal state of motion inside the reservoir. All information about the m→n quantum transition, therefore, is once again lost, which means that Figure 3c represents another case of irreversible and unobservable mechanical motion.

**Figure 5:**
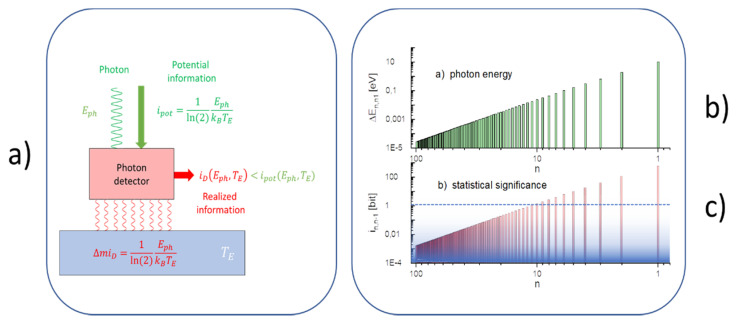
(**a**) Photon detector turning potential information $i_{pot}$ into missing information $mi_{D}$ and transiently producing macroscopically observable events with informational value $i_{D}<i_{pot}$; (**b**) photon cascade produced by de-excitation of a highly excited H-atom, de-exciting from a quantum state with m=100 toward its ground state with n=1 with a step size of Δn=1; (**c**) potential information carried with the emitted photons and measured relative to the detector temperature of $T_{E}=300\;K$. The graded blue background indicates the impact of thermal detector noise, with the limit of $i_{pot}=1\;bit$ denoting a conventional signal-to-noise ratio of SN=1.

Figure 3d, finally, looks very similar to Figure 3c. In this latter example, however, the passively absorbing reservoir walls of Figure 3c are replaced by walls which are able to perform as photon detectors. Like the passive walls in Figure 3c, the detector walls again absorb all photons that hit their inner surfaces and that dissipate their energies $\Delta E_{mn}$ at the wall temperature $T_{E}$. During dissipation, however, these detector walls function as thermodynamic engines which transiently convert the dissipated energy into macroscopically observable events that can be monitored at their outside surfaces [9]. As shown in Figure 5a and discussed in more detail in the Appendix A, these events constitute pieces of physical action endowed with an observational value $i_{D}\left(E_{ph},T_{E}\right)<i_{pot}\left(E_{ph},T_{E}\right)$, which measures the probability that an observed event is caused by a true photon–detector interaction and unlikely by a random thermal excitation inside the detector wall itself. Figure 3d, therefore, once again represents an open system, but one endowed with the capability of providing macroscopically observable images at its outside surfaces of those microscopic mechanical processes that are going on within the reservoir walls.

Whereas a single m→n transition produces as a mirror image, a single macroscopically observable event, i.e., an elementary observation, a highly excited H-atom that was initially excited to a stationary state with quantum number m=100 and subsequently allowed to relax back to its ground state with n=1, lowering in each step the final-state quantum number by one unit, produces a more complex observational image. While Figure 5b shows the photon cascade that results from such an emission process, Figure 5c plots the levels of potential information that are carried with each photon toward the detector walls. As these latter values represent upper limits to the informational value $i_{D}<i_{pot}$, which might be gained in a detection process, the bottom panel clearly shows that the majority of detection events at the beginning of the cascade, with $i_{pot}<1\;bit$, actually become buried inside the detector noise as the corresponding photons have energies lower than the mean thermal energy inside the detector walls.

Whereas the effects of detector noise can simply be reduced by lowering the detector operation temperature, another limiting factor is detector response time. When detector response times become long compared to the radiative lifetimes of the excited quantum systems, successive emission events might pile up with the piled-up responses ultimately looking like continuously varying responses produced by classical radiation fields. Unlike the effects of thermal noise, which can simply be combatted by reducing detector operation temperatures, the temporal broadening of detector output signals needs to be tolerated to a certain extent to make microscopic photon–detector interactions observable at a macroscopic scale. This latter effect is explained in more detail in the Appendix A.

## 4. Least Action Principles and Observational Overhead

The above discussion has shown that the emission of radiation from moving charged particles adds an element of irreversibility both on their classical and quantum mechanical motions. While the emission of each photon from a charged particle represents an interaction event in which mechanical energy is withdrawn from the moving particle, the emitted photon in turn is able to trigger a follow-on event in which the photon energy is dissipated and a macroscopically observable event is generated, which makes the mechanical motion observable from a distance. As both emission and detection events represent pieces of physical action (see Appendix A), which add onto the physical action of the particle motion itself, it becomes evident that the emission of radiation and its eventual detection represent overheads on the particle motion that are measurable in units of physical action. Below, we present evidence that the principle of least action, which is at the heart of Hamilton’s equations of motion, not only minimizes the physical action that is associated with the particle motion itself but that it also tends to minimize those dissipative and observational overheads that are associated with the physically realized trajectories.

**Figure 6:**
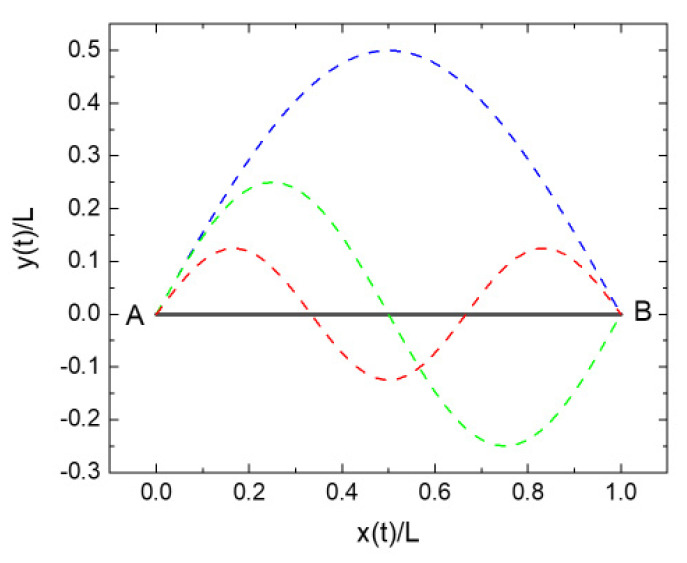
Possible trajectories of a particle moving from points A to B in the absence of any external force fields: full line, physically realized motion; dashed lines, alternative routes connecting A and B but prohibited by the principle of least action.

In order to demonstrate this, consider the example presented in Figure 6. There, a charged particle of mass M is supposed to move from the far left-hand to the far right-hand side of this graph, following a straight-line path in the absence of any external force fields. On its way, the particle also passes through the line section $L=\vec{AB}$. Figure 6 also shows several alternative routes, connecting points A and B, which the particle might take but which are ruled out by the principle of least action. In assessing this situation, we firstly calculate the physical action that is associated with the particle motion from point A to B itself, ignoring for the moment the emission of electromagnetic radiation. In a second step, we determine the overhead in physical action that is associated with the emission of electromagnetic radiation that occurs as the particle follows the sinusoidal alternative routes.

In order to calculate the associated pieces of physical action, we firstly parameterize the different trajectories in Figure 6. Assuming that the particle moves with speed $v_{x}$ along the *x*-axis of our coordinate system, covering the distance L between points A and B in time $\tau =L/v_{x},$ the *x*-coordinates of all trajectories become

(20) $$x\left(t\right)=v_{x}t,\;0\;\leq \;t\;\leq \;\tau .$$

Allowing additional excursions into the *y*-direction, we put

(21) $$y\left(a,n,t\right)=a\sin\left[n\pi \left(\frac{v_{x}}{L}\right)t\right],n=0,\;1,\;2,\;3\ldots ,$$

where a stands for the amplitude of excursion, and n stands for the number of excursions as the particle moves form A and B.

With these coordinates, it is easy to calculate the kinetic energy of the particle as it moves along the distance L and the physical action generated upon arrival at point B.

(22) $$E_{kin}\left(t\right)=\frac{1}{2}M\left[{\dot{x\left(t\right)}}^{2}+\dot{y{\left(a,n,t\right)}^{2}}\right],$$

(23) $$W_{mech}\left(a,n,\tau \right)=\int_{0}^{\tau }E_{kin}\left(t\right)\;dt=\frac{1}{2}Mv_{x}{}^{2}\tau \;\left[1+\frac{1}{2}n^{2}\pi^{2}{\left(\frac{a}{L}\right)}^{2}\right].$$

This latter result shows that $W_{mech}\left(a,n,\tau \right)$ takes on a minimum value in case the number of excursions n and/or their relative amplitudes a/L are minimized, i.e., in case a straight-line motion is followed.

We now move on to a particle carrying an electrical charge Q and performing the same trips as before. In case the charged particle makes excursions into the *y*-direction, it suffers accelerations and, thus, emits electromagnetic radiation as predicted by the Larmor formula [10,11,17,18].

(24) $$P_{rad}\left(a,n,t\right)=\frac{Q^{2}}{6\pi \varepsilon_{0}c^{3}}{\left(\frac{d^{2}y\left(a,n,t\right)}{dt^{2}}\right)}^{2}$$

where $\varepsilon_{0}$ stands for the vacuum dielectric constant and c for the speed of light. With this formula and the coordinate functions above, the electromagnetic energy $E_{rad}$ and the associated physical action $W_{rad}$ can be evaluated by double integration of Equation (24) over the time duration of travel τ. In this way, one obtains

(25) $$E_{rad}\left(a,n,\tau \right)=\frac{1}{3}{\left(\frac{Q}{q}\right)}^{2}\alpha_{FS}\hbar {\left[n^{2}\pi^{2}\left(\frac{a}{L}\right)\right]}^{2}{\left[\frac{v_{x}}{c}\right]}^{2}\frac{1}{\tau }$$

(26) $$W_{rad}\left(a,n,\tau \right)=E_{rad}\left(a,n,\tau \right)\;\tau$$

(27) $$W_{rel}\left(a,n,\tau \right)=\frac{W_{rad}\left(a,n,\tau \right)}{W_{mech}\left(a,n,\tau \right)}=\left[\frac{n^{4}\pi^{4}{\left(\frac{a}{L}\right)}^{2}}{1+\frac{1}{2}n^{2}\pi^{2}{\left(\frac{a}{L}\right)}^{2}}\right]{\left(\frac{Q}{q}\right)}^{2}\left(\frac{m_{e}}{M}\right)\left(\frac{\tau_{rad}}{\tau }\right)$$

In these latter equations, $\alpha_{FS}$ stands for the fine-structure constant,

(28) αFS=q24πε0ħc≈1137

while

(29) $$\tau_{rad}=\frac{2}{3}\alpha_{FS}\frac{\hbar }{m_{e}c^{2}}\approx 10^{-23}s$$

stands for the radiation time constant of the electron [10].

Returning to Equation (27), it can be seen that not any dissipative overhead arises in case the particle follows a straight-line path as predicted by Hamilton’s equations of motion. With the principle of least action obviously preferring trajectories with minimum curvature, it is thereby suggested that the principle of least action not only minimizes those pieces of physical action that are associated with the particle motion itself but also those that are associated with the emission of electromagnetic radiation. With the effects of radiation emission being minimized, it also follows that any follow-on effects such as the dissipation of the radiation energy in the environment and its eventual detection in natural (eye) or man-made radiation detectors are minimized. From the standpoint of thermodynamics and information theory, the principle of least action therefore appears as a principle of minimum entropy production and/or minimum information gain.

Interestingly, such behavior also agrees with the results of Bormashenko [20,21], who analyzed the recording and erasure of information carried by particles trapped inside a minimum Szilard-type engine under the influence of externally applied inertial forces. The attractive aspect of this latter approach is that it allows informational principles of mechanical motion to be extended to neutral particles, which do not directly couple to electromagnetic fields.

## 5. Dissipative Overhead and Irreversibility

In the example of Figure 6, we tacitly assumed that the particle motion occurs in free space, i.e., with the particle avoiding any collisions with other pieces of matter as it proceeds from points A to B. A very different situation arises when the particle is a molecular ion that is moving through a gas of neutral molecules with the same mass. With gas–kinetic collisions now taking place, the ion is forced into many of those small excursions from a straight-line path which were previously prohibited by the principle of least action. In short, the particle then no longer follows a zero-dissipation path as in the example of Figure 6 above.

Inside such a gas, the ion suffers frequent and violent ion–molecule interactions in which the ion becomes accelerated or decelerated and in which it is forced to emit bursts of electromagnetic radiation. Once emitted, the electromagnetic radiation either runs away from its sites of emission and disappears in empty space, as in the scenario of Figure 2b, or it becomes absorbed inside a container wall, as in Figure 2c. Whereas, in the first case, some of the motional energy is extracted from the moving ion and irreversibly dispersed in space, the radiation energy absorbed inside one of the container walls is converted into thermal radiation and re-emitted into the gas where it eventually might become re-absorbed by the moving ion once again. As, in both cases, the resulting motional changes do not conform with the mathematical time reversal operation t→−t, both situations obviously involve a certain degree of irreversibility.

**Figure 7:**
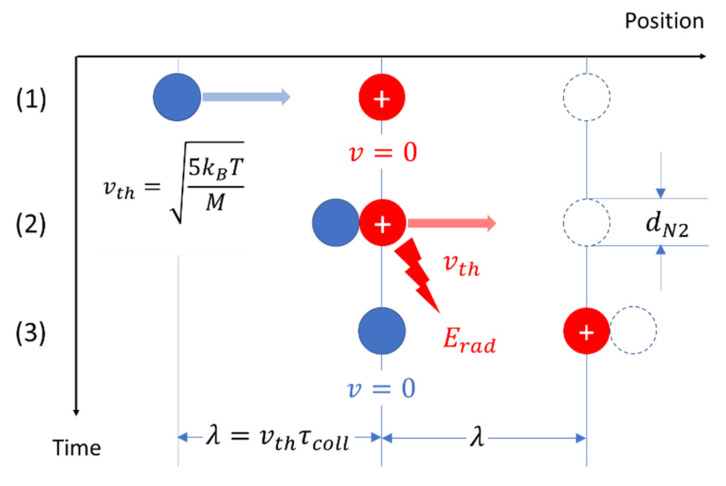
Molecule–ion interaction inside a gas of temperature T leading to an exchange of motional energy between the molecule and ion and causing the emission of a pulse of electromagnetic radiation upon interaction (2).

In order to determine the amount of irreversibility that might be involved in a gas–kinetic collision, consider Figure 7. There, a neutral N_2_ molecule is assumed to be coming from the left (1), moving with the mean thermal velocity $v_{th}=\sqrt{5k_{B}T/M}$, hitting an $N_{2}{}^{+}$ ion in the center (2), which is initially at rest, and accelerating it during the time $\tau_{int}=d_{N2}/v_{th}$ from v=0 to $v=v_{th}$, thus moving the ion by one mean-free distance to the right and bringing the neutral molecule to rest at the former position of the $N_{2}{}^{+}$ ion (3).

Using the Larmor radiation formula (Equation (24)), for the radiation energy that is emitted from each site of interaction, one obtains

(30) $$E_{rad}\left(\tau_{int}\right)=2\sqrt{3}\;\alpha_{FS}\hbar \left(\frac{c}{d_{N2}}\right){\left(\frac{k_{B}T}{Mc^{2}}\right)}^{3/2}$$

With this result, the following important parameters can be calculated:

(i) the fractional loss of mechanical energy, $\delta E_{rad}\left(\tau_{int}\right)=E_{rad}\left(\tau_{int}\right)/E_{th}$, in each ion–molecule interaction,
(ii) the number of collisions $n_{diss}=1/\delta E_{rad}$ required to transfer the mean thermal energy of the moving ion toward its environment where it becomes dissipated,
(iii) the total time, $\tau_{diss}=n_{diss}\tau_{coll}$, required for this energy transfer,
(iv) the length of the diffusion path, $L_{diss}=\sqrt{\left(\frac{1}{6}\frac{\lambda^{2}}{\tau_{coll}}\right)\tau_{diss}}$, covered in the time $\tau_{diss}$.

Assuming standard temperature–pressure conditions for the gas, these values are listed in Table 1.

Looking at the data in row 1, the assumption has been made that the ion as a whole is emitting the radiation. Due to its relatively large mass of $M_{ion}=28\;amu$, a very small amount of radiation is emitted with the consequence that an extremely large number of gas–kinetic collisions is required to completely convert the mean thermal energy of the ion into radiation energy. Consequently, very long dissipation times $\tau_{int}$ and diffusion lengths $L_{diss}$ arise.

In view of this result, the assumption has been made in row 2 that the gas–ion interactions are not with the ion as a whole but with electrons either bound to the ion itself or to its neutral collision partners. In this way, much larger energy transfers per interaction occur and a smaller number of interactions $n_{diss}$ and smaller length and time scales $L_{diss}$ and $\tau_{diss}$ result. Both of these length and time scales are clearly of macroscopic size but much longer than the length and time scales of the individual ion–molecule interactions $d_{N2}$ and $\tau_{int}=d_{N2}/v_{th}$, respectively. Furthermore, involving electrons in the process of radiation damping and energy dissipation involves the attractive aspect that such interactions in principle can also appear in interactions between neutral molecules. A certain drawback of our classical Larmor formula approach to radiation damping and energy dissipation is the small amounts of radiation energy of about $10^{-7}\mathrm{eV}$ that are assumed to be emitted in each ion–molecule interaction which correspond to typical radio frequencies. A full quantum-mechanical treatment of gas–kinetic processes much more likely will yield emission energies on the order of the rotational quantum energies of N_2_ molecules and, concomitantly, much smaller emission probabilities during each gas–kinetic interaction. With this caveat in mind, we assume that the bottom line of results in Table 1 reasonably approximates the real level of irreversibility in the gas–kinetic process.

## 6. Summary and Conclusions

Above, we discussed the subject matter of mechanical motion of charged particles with a special emphasis on detection. A key enabling effect for the detection of mechanical motion is the process of radiation damping. With the emitted radiation extracting mechanical energy from the moving particles and dispersing it in the environment, radiation damping introduces an element of irreversibility into the mechanical motion. Furthermore, as the emission represents an event that constitutes a piece of physical action, it becomes clear that the process of radiation damping can be regarded as a dissipative overhead that is burdened onto the mechanical motion and that is measurable in units of physical action and entropy.

With the emitted radiation carrying away signatures of those changes in mechanical motion that had caused its emission, the emitted radiation, in principle, makes mechanical motion detectable from a distance. This is particularly evident in the case of photons emitted from excited atoms. In this case, the changes in electronic orbital energy and in orbital angular momentum inside an atom are encoded into the energy and the angular momentum of the emitted photons. With the photons carrying these signatures away from their sites of emission to potential sites of observation, photons are obvious carriers of potential information. In order to make the transported information available at the sites of observation, the photon energy needs to be dissipated there and a macroscopically observable event has to be formed that represents an amplified version of the microscopic emission event. Such amplification features in the form of a huge increase in the physical action of detection ($W_{D}$) over the physical action of emission (ħ) and an associated elongation of the detector response times ($\tau_{D}$) over the time duration of the emission event ($\tau_{em}$) (see Appendix A) [9]. Furthermore, as the detection process involves energy dissipation and thermal noise, detection events represent noisy and temporally elongated images of the respective emission events. With observation following emission, observation once more appears as a dissipative overhead that is burdened onto the mechanical motion and that needs to be accepted in order to allow mechanical motion to be actually observed.

With observation being related to physical action and with observational overheads adding onto the physical action of the mechanical motion itself, both overheads should be controlled by the principle of least action. Considering an example of constant-speed rectilinear motion in the absence of any external force fields, we have shown that the principle of least action not only minimizes the physical action of the mechanical motion itself but that it also minimizes any overheads that might be associated with the radiation damping and the eventual detection of the emitted radiation. In the example considered, this means that constant-speed rectilinear motion is unobservable and fully reversible, in full agreement with Hamilton’s equations of motion. Extrapolating from this result, we propose that the principle of least action, which is at the heart of Hamilton’s equations of motion, could also be viewed as a principle of minimum energy dissipation and/or of minimum information gain.

Quantitative estimates of the dissipative burdens on mechanical motion show that the effects of radiation damping are close to immeasurably small as long as the mechanical motion of macroscopic particles at non-relativistic speeds is concerned. In the case of swiftly moving and intensely interacting particles, such as molecules moving inside macroscopic volumes of gas, the dissipative burdens can take on measurable sizes, thus shedding some doubt on the assumed ideal reversibility of mechanical motion at the molecular scale.

## Figures and Tables

**Table 1:** Relative loss of motional energy $\delta E_{rad}\left(\tau_{int}\right)$ due to radiation damping during gas–kinetic collisions, number of collisions $\left(n_{diss}\right)$ required for a complete transfer of the mean thermal ion energy into radiation, total time required for a complete energy transfer ($\tau_{diss}$), and length of ionic diffusion path expected to be covered during time $\tau_{diss}$ ($L_{diss}$).

Interaction	$\delta E_{rad}\left(\tau_{int}\right)$	$n_{diss}$	$\tau_{diss}\;\left(s\right)$	$L_{diss}\;\left(\mathrm{cm}\right)$
Gas–ion	$6.90\times 10^{-16}$	$1.45\times 10^{15}$	$1.95\times 10^{6}$	$1.08\times 10^{3}$
Gas–electron	$7.97\times 10^{-9}$	$1.26\times 10^{8}$	0.17	0.32

**Figure A1:**
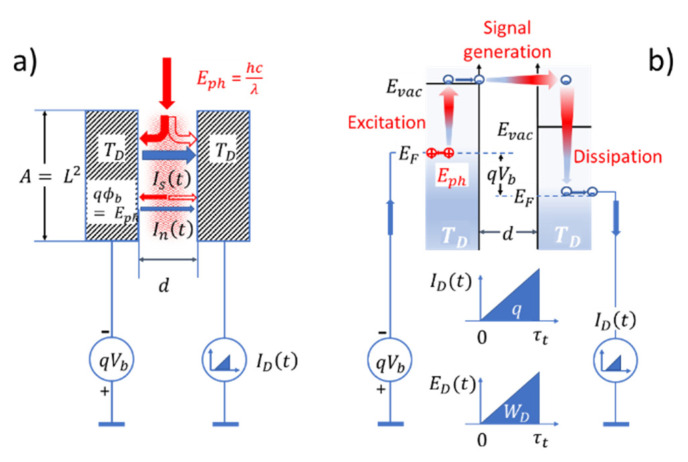
(**a**) Signal photon (red vertical arrow) entering a gap between two metal plates. Photons absorbed inside the negatively biased emitter electrode release signal photoelectrons (blue horizontal arrows). Emitter and collector electrodes maintained at the ambient temperature $T_{D}$ produce blackbody radiation (red cloud), producing noise electrons. Signal and noise currents together produce externally observable detector currents $I_{D}\left(t\right)$; (**b**) processes of photoelectron excitation, signal generation, and energy dissipation as seen in a band diagram.

## Appendix A. Photon Detection

Here, we summarize the key results of a previous paper [9] in which we showed that detection events represent pieces of physical action produced at the expense of dissipation of photon energy and endowed with an observational value, which measures the probability that the events were produced by true photon–detector interactions and unlikely by random thermal fluctuations inside the detector itself.

In order to see how energy dissipation can be put to work to generate pieces of realized information, we consider the process of photon detection in a photoionization detector (PID). The reason for choosing the example of PIDs is that these are conceptually simple devices and that these most closely resemble ideal photon detectors [9,16]. Photon detection in PIDs was already treated in some detail in a previous publication [9]. Here, we briefly summarize the main results as far as these are relevant in the present context. PIDs are basically parallel-plate capacitor devices consisting of metal plates with a work function $q\phi_{m}$ and biased to a static potential $V_{b}$ (Figure A1). When photons with energy $E_{ph}\geq \;q\phi_{m}$ enter the gap in between both plates, electrons can be excited to the vacuum levels in both plates, thereby enabling them to enter the gap in between both plates. When electrons are released from the negatively biased emitter electrode, triangular electrical current pulses,

(A1) $$I_{s}\left(t\right)=2\;q\frac{t}{\tau_{t}{}^{2}}\;\left(0\leq t\leq \tau_{t}\right)$$

are induced, which abruptly end when the photoelectrons reach the grounded collector electrode at times $\tau_{t}$, where the current flow can be monitored.

The current pulses (Equation (A1)), obviously, are observational images of those microscopic photon–detector interactions in which photons are converted into photoelectrons. These current pulses, therefore, represent pieces of realized information. Considering the fact that these current transients have a finite time duration $\tau_{t}$ and that these occur within the finite volume of the detector gap, these pieces of information obviously take the form of space–time events. In addition to space and time coordinates and space–time extension, such detection events are endowed with additional properties, which are revealed from integrals taken over the time duration of these events.

A first and obvious measure is the collected charge,

(A2) $$q=\int_{0}^{\tau_{t}}I_{s}\left(t\right)dt$$

A second important measure can be obtained by multiplying the current pulses $I_{s}\left(t\right)$ with the potential drop through which the photoelectrons fell on their travel through the detector gap. In this way, the signal power $P_{s}\left(t\right)$ is obtained. Double integration over time then successively leads to the signal energy $E_{s}\left(t\right)$ and the physical action $W_{s}\left(t\right)$ after a time t and, finally, the physical action obtained after termination of the current transient,

(A3) $$W_{D}\left(\tau_{t}\right)=\frac{1}{3}qV_{b}\tau_{t}$$

This latter result shows that detection events are not merely quadruples of space-time coordinates, but pieces of physical action. More importantly, the pieces of physical action $W_{D}\left(\tau_{t}\right)$ represent a physically measurable value that was gained in the detection process.

Once a photoelectron arrives at the collector electrode, its kinetic energy is dissipated inside this electrode, i.e., broken up into pieces of energy of size $k_{B}T_{D}\ll qV_{b}$ and, therefore, converted into low-temperature heat. Due to the thermal coupling of the PID to the environment, this low-temperature heat ultimately ends up as entropy transferred to the environment,

(A4) $$S_{D}=\frac{E_{ph}+qV_{b}}{T}$$

As the photoelectron energy is negligible in comparison to the huge internal energy U of the environmental reservoir ($U\gg E_{ph}+qV_{b}$), a measurable change in the environmental temperature T does not result, and the entropy $S_{D}$ finally ends up as missing information,

(A5) $$mi_{D}=\frac{1}{ln\left(2\right)}\frac{E_{ph}+qV_{b}}{k_{B}T},$$

concerning the internal state of motion inside the huge environmental reservoir. This conversion of photoelectron energy into low-temperature heat, obviously, represents the physical price aid for transiently obtaining the piece of physical action $W_{D}\left(\tau_{t}\right)$. Eliminating $qV_{b}$ from Equations (A3) and (A4), a simple proportionality between physical action gained and entropic price paid is obtained,

(A6) $$W_{D}\left(\tau_{t}\right)=\frac{1}{3}\left(\tau_{t}T\right)S_{D}\left(T\right)$$

Interestingly, this proportionality depends on the time duration $\tau_{t}$ that the electron travel through the gap took. Physically, this time duration is the minimum time that is required to reset the detector to its pre-detection state and to ready it for a new round of photon detection. As shown in our previous paper [9], this time duration depends on the detector gap width and the bias potential applied across this gap,

(A7) $$\tau_{t}\left(d,V_{b}\right)=\frac{1}{2}\tau_{ph}\left(\frac{d}{d_{min}}\right)\sqrt{\frac{2m_{e}c^{2}}{qV_{b}}}$$

with $\tau_{ph}$ standing for the photon vibrational period and $d_{min}=\lambda /2$ standing for the minimum detector gap width which corresponds to half the photon wavelength $\lambda =hc/E_{ph}$; $m_{e}c^{2}$, finally, is the electron rest mass. In case $qV_{b}$ is chosen to match the photon energy $E_{ph}$ and d is the minimum gap size of $d_{min}=\lambda /2$, this reduces to

(A8) $$W_{D}\left(\tau_{t}\right)=\frac{1}{6}h\sqrt{\frac{2m_{e}c^{2}}{E_{ph}}}\gg \hbar$$

which shows that the physical action gained upon detection hugely exceeds the physical action carried with the photon prior to its detection in the form of its spin angular momentum. The inequality in Equation (A8), therefore, shows that detection involves a fair bit of amplification, which results from the elongation of the electron transit time $\tau_{t}$ to time scales much larger than the photon vibrational period $\tau_{ph}=h/E_{ph}$. Such an elongation is instrumental in turning microscopic interaction events into macroscopically observable events. Large values of $\tau_{t}$, however, limit the rate with which photons can be counted.

As a final point, we note that every detection event only has a finite observational value, as detection events, in principle, can also arise from photons, thermally generated within the detector itself (see Figure A1). As shown in our recent paper [9], the statistical significance of detection events can be measured by the magnitude of the information realized in the detection process, which is related to the classical signal-to-noise ratio, $SN\left(E_{ph},T\right)$, by

(A9) $$i_{real}\left(E_{ph},T\right)=\frac{1}{ln\left(2\right)}ln\left(SN\left(E_{ph},T\right)\right)\leq i_{pot}\left(E_{ph},T\right)=\frac{1}{ln\left(2\right)}\frac{E_{ph}}{k_{B}T}.$$

As shown there, $i_{real}\left(E_{ph},T_{D}\right)$ hardly ever exceeds values of $\sim 0.5\;i_{pot}\left(E_{ph},T_{D}\right)$, i.e., half the potential information carried with the photons prior to detection and relative to a detector operated at a temperature T.
